# Assessing the influence of maternal vitamin D deficiency in early pregnancy and subsequent improvement on perinatal outcomes and long-term child development: a retrospective cohort study

**DOI:** 10.1371/journal.pone.0323146

**Published:** 2025-05-30

**Authors:** Ji Yeon Lee, Sang Hee Jung, Eun Hee Ahn, Hyun Mee Ryu

**Affiliations:** Department of Obstetrics and Gynecology, CHA Bundang Medical Center, CHA University School of Medicine, Seongnam, Republic of Korea; Universiti Sains Malaysia, MALAYSIA

## Abstract

This study examined perinatal and long-term outcomes where maternal vitamin D deficiency was present in early pregnancy but normalized in mid-pregnancy and deficiency during both early and mid-pregnancy. In this retrospective study, we reviewed the clinical records of 5,169 singleton pregnant women who received a test of serum 25-hydroxy-vitamin D [25(OH)D] two times in the first and second trimester in our hospital from 2016 to 2022. The level of 25(OH)D was categorized into deficiency (<10ng/mL), insufficiency (10–20ng/mL), and sufficiency (≥20ng/mL). Women were grouped based on 25(OH)D status across both trimesters: consistently deficient (DD), initially deficient then sufficient in the second trimester (DS), and consistently sufficient (SS). We evaluated obstetric and neonatal outcomes, including long-term neurodevelopmental assessments. Measurements in the first trimester indicated that 21.7% (n = 1,120) of women were vitamin D deficient, 41.1% (n = 2,127) insufficient, and 37.2% (n = 1,922) sufficient. There were no significant differences in the incidence of gestational hypertension and diabetes among the DD, DS, and SS groups. The rates of preterm birth before 34 weeks (aOR = 2.42, 95%CI[1.24–4.71], p = 0.010), necrotizing enterocolitis (aOR = 22.26, 95%CI[4.16–119.34], p < 0.001), and developmental delay (aOR = 4.46, 95%CI[2.41–8.27], p < 0.001) were elevated in the DD group compared to the SS group. These risks didn’t diminish even when vitamin D deficiency in the second trimester was corrected (DS). In conclusion, associations between low first-trimester 25(OH)D levels and heightened risks of preterm birth and long-term developmental outcomes are observed. As subsequent normalization of 25(OH)D levels may not fully mitigate these risks, incorporating vitamin D screening and intervention before pregnancy as part of routine preconception care could be beneficial in optimizing maternal and offspring outcomes.

## Introduction

Vitamin D, a hormone crucial for maintaining calcium and phosphorus homeostasis, plays a pivotal role in various physiological processes [1]. Among its multifaceted functions, vitamin D has gained increasing attention for its potential impact on a healthy pregnancy and normal development of the fetus [2]. A recent review suggested that maternal serum 25-hydroxyvitamin D [25(OH)D] levels should be at least 40 ng/mL to optimize both maternal and fetal health [3]. While the concentration optimal cutoff point for vitamin D deficiency may vary based on factors such as health status, age, and lifestyle, several organizations, including the World Health Organization, generally define vitamin D deficiency as a serum 25-hydroxy-vitamin D [25(OH)D] concentration below 20 ng/mL [4–6].

Vitamin D deficiency during pregnancy has been linked to risks of gestational diabetes, gestational hypertension, and preeclampsia [7,8]. Previous studies reported a potential connection between maternal vitamin D deficiency and an increased risk of preterm birth and small for gestational age offspring. Additionally, insufficient maternal vitamin D levels may impact fetal skeletal development, potentially leading to conditions like rickets. Separately, maternal vitamin D deficiency has also been associated with adverse neonatal outcomes, such as respiratory distress syndrome (RDS) and neonatal hypocalcemia [9–11]. Furthermore, some studies have shown potential associations between maternal vitamin D deficiency and an elevated risk of neurodevelopmental disorders in the offspring such as cognitive impairment, and behavioral issues [12–14].

A 2016 systematic review reported maternal vitamin D deficiency rates of 64% in the Americas, 57% in Europe, 46% in the Eastern Mediterranean, 87% in Southeast Asia, and 83% in the Western Pacific [14]. In South Korea, the situation is even more concerning, with studies indicating that approximately 80–86% of Women of childbearing age have insufficient or deficient vitamin D levels [6]. Despite the widespread prevalence of vitamin D deficiency among pregnant women across diverse populations and ethnic groups, its impact on obstetric complications and long-term outcomes for both mothers and infants remains an area of ongoing investigation.

Moreover, critical challenges persist in addressing vitamin D deficiency during pregnancy, including inadequate awareness among healthcare providers and pregnant women about the importance of maintaining optimal vitamin D levels. Limited routine screening for maternal vitamin D status during pregnancy further impedes timely interventions. The problem is further complicated by the fact that while vitamin D can be synthesized endogenously through skin exposure to ultraviolet B radiation, various lifestyle, cultural, religious, occupational, and gender-related factors can significantly limit sun exposure. Although dietary sources and fortified foods contribute to vitamin D intake, their impact is often negligible, making it challenging for pregnant women to maintain adequate vitamin D levels without sufficient sun exposure or supplementation [15].

Previous studies emphasize the importance of adequate vitamin D levels during pregnancy. However, a comprehensive understanding of how maternal vitamin D deficiency may impact perinatal and long-term outcomes is still evolving. Specifically, the critical period in pregnancy at which vitamin D deficiency becomes a significant concern remains unclear. The absence of studies addressing whether vitamin D optimization should commence before pregnancy or if starting in the first trimester is too late further complicates this understanding. Therefore, this study aimed to examine perinatal and long-term outcomes in cases where vitamin D deficiency was present in early pregnancy but normalized in mid-pregnancy through appropriate intervention, as well as maternal vitamin D deficiency or sufficiency during both early and mid-pregnancy.

## Materials and methods

### Patients and study design

In this retrospective cohort study, we included women with singleton pregnancies who visited CHA Bundang Medical Center from March 2016 to December 2022. Cases with multifetal pregnancies, maternal medical or surgical problems before pregnancy, or chromosomal abnormalities in the fetus were excluded.

**Fig 1:**
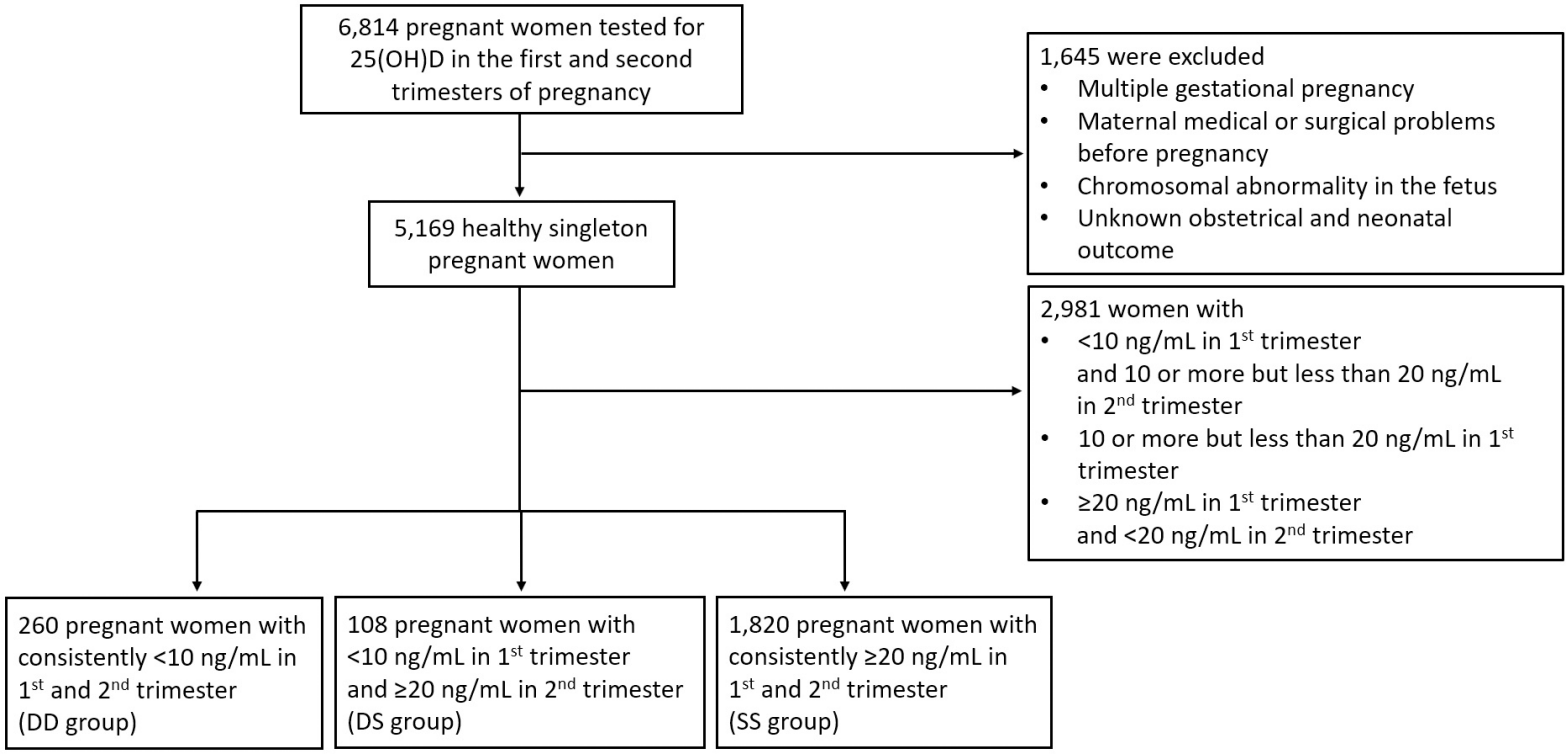
Diagram showing the flow of study selection and group allocation.

At our center, maternal serum vitamin D levels are routinely measured as part of standard antenatal blood tests during both the first and second trimesters. Serum 25(OH)D concentrations were assessed twice during pregnancy: once in the first trimester and again in the second trimester, at varying times within each trimester. The mean gestational age at the time of the first-trimester measurement was 11.2 ± 1.4 weeks in the DD group, 11.5 ± 1.1 weeks in the DS group, and 11.2 ± 1.4 weeks in the SS group (P = 0.178). For the second-trimester measurement, the mean gestational ages were 22.5 ± 2.8 weeks, 22.3 ± 3.2 weeks, and 22.3 ± 3.0 weeks, respectively (P = 0.792). These measurements were categorized into three distinct groups based on widely accepted clinical thresholds: deficiency (<10 ng/mL), insufficiency (10–20 ng/mL), and sufficiency (≥20 ng/mL) [16]. Women were further categorized into three groups based on their vitamin D status across both trimesters: the consistently deficient group (DD), the initially deficient then sufficient post-intervention group (DS), and the consistently sufficient group (SS) (Fig 1).

After assessing serum 25(OH)D concentrations in the first trimester, pregnant women received counseling from their healthcare providers regarding the significance of adequate vitamin D intake throughout pregnancy, both for their well-being and the optimal development of their infants. The counseling provided recommendations: 1) Integrate vitamin D-rich foods like fish, dairy products, and eggs into your daily diet. 2) Opt for moderate sun exposure, as it stimulates the skin’s production of vitamin D. 3) If ensuring adequate vitamin D intake through diet and sunlight exposure is difficult, contemplate vitamin D supplementation (approximately 1000 IU).

### Ethics approval

The institutional review boards (IRB) of CHA Bundang Medical Center approved this study (IRB no.: 2024-03-029). Informed consent was waived for this retrospective cohort study as it involved the analysis of medical records. The IRB of the research institute approved the study and determined that obtaining informed consent was not necessary. The study methods strictly adhered to the relevant guidelines and regulations set forth by the IRB at this institution. The period for which data was accessed for research purposes is from March 29, 2024 to October 31, 2024. The medical records of patients included in this study are stored in the Case Report Form (CRF), and the patient names and medical history numbers are encrypted in the CRF so that individual participants cannot be identified. Therefore, the authors can’t access information that can identify individual participants during or after data collection.

### Serum 25(OH)D levels measurement

The 25(OH)D level was measured as follows: Maternal venous blood samples were collected and centrifuged at 3500 g for 10 minutes at 4 °C to obtain the serum. They were promptly transferred and stored in aliquots at -80 °C until biochemical analysis could be conducted. Serum 25(OH)D levels were quantified using a commercially available kit and chemiluminescence immunoassay, following the manufacturer’s protocol (LIAISON 25 OH Vitamin D TOTAL Assay REF 310600).

### Review of medical records

We reviewed obstetric characteristics, including maternal age, body mass index (BMI) before pregnancy, parity, method of conception, and relevant medical conditions during pregnancy such as gestational hypertension, diabetes, and placenta previa. Additionally, perinatal outcomes, such as transient tachypnea of the newborn (TTN), RDS, bronchopulmonary dysplasia (BPD), jaundice, neonatal enterocolitis (NEC), and intracranial hemorrhage (ICH), were reviewed. We investigated long-term neurodevelopmental outcomes after one year of corrected age, focusing on the diagnosis of developmental delay, which was defined as the failure to achieve developmental milestones at the expected times. Follow-up assessments were conducted up to the oldest age at which each child was evaluated during the study period. To assess this, we used the Bayley Scales of Infant and Toddler Development, Third Edition (Bayley-III), and/or the Gross Motor Function Measure. Developmental delay was identified when test scores fell below the reference range for normal development. In cases where formal developmental screening tests were not performed, we reviewed medical records to gather information on the child’s developmental status. Healthcare providers conducted brief developmental assessments and used age-appropriate questionnaires covering language, cognition, behavior, and motor skills, documenting any concerns. A child’s development was considered normal if there were no reported difficulties in meeting academic expectations.

### Statistical analysis

Statistical analysis utilized SPSS software (version 28.0; SPSS Institute, Chicago, IL, USA). Differences between groups were assessed using the Chi-square test for categorical variables and one-way ANOVA, followed by Bonferroni’s post-hoc comparison test for continuous variables. Additionally, multivariate analyses were performed using explanatory variables to calculate adjusted odds ratios (aORs). Significant explanatory variables identified in univariate analysis, along with clinically important variables including maternal age, pre-pregnancy BMI, parity, and method of conception, were entered into a multivariate model. Statistical significance was established at a p-value less than 0.05.

## Results and Discussion

A total of 5,169 healthy singleton pregnant women were included in this study. Initial measurements in the first trimester revealed that 21.7% (n = 1,120) were vitamin D deficient, 41.1% (n = 2,127) insufficient, and 37.2% (n = 1,922) sufficient.

Among the 1,120 patients initially deficient in 25(OH)D during the first trimester, 260 patients remained deficient (<10 ng/mL) in the second trimester (DD group), while 108 patients improved to sufficiency (≥20 ng/mL). When compared with the consistently sufficient (SS) group of 1,820 women, significant differences emerged in maternal age, with the DD group at 33.8 ± 4.1 years, the DS group at 34.2 ± 4.1 years, and the SS group at 35.0 ± 3.9 years (p < 0.001). The rate of nulliparity was higher in both the DD group (86.9%) and the DS group (90.7%) compared to the SS group (82.4%) (p = 0.019). Additionally, more cases of pregnancies through the In Vitro Fertilization (IVF) and Embryo Transfer method were noted in the SS group (49.6%) than in the DD group (24.2%) and the DS group (14.8%) (p < 0.001). However, no significant differences were observed in BMI, parity, history of preterm birth, and method of conception among the three groups (Table 1).

**Table 1:** Clinical characteristics of the study population.

	DD group (N = 260)	DS group (N = 108)	SS group (N = 1820)	*p*-value^a^	*p*-value^b^	*p*-value^c^	*p*-value^d^
Maternal age (year)	33.8 ± 4.1	34.2 ± 4.1	35.0 ± 3.9	<0.001	<0.001	0.121	0.964
BMI in pre-pregnancy	22.8 ± 3.7	22.6 ± 3.8	22.5 ± 3.7	0.534	0.825	1.000	1.000
Nulliparity	226 (86.9)	98 (90.7)	1499 (82.4)	0.019			
Prior preterm birth	4 (1.5)	0 (0.0)	30 (1.6)	0.404			
Method of conception				<0.001			
Spontaneous	183 (70.4)	87 (80.6)	814 (44.7)				
Ovarian stimulation	14 (5.4)	5 (4.6)	104 (5.7)				
In vitro fertilization	63 (24.2)	16 (14.8)	902 (49.6)				
25(OH)D in the first trimester (ng/mL)	7.4 ± 1.8	7.6 ± 1.8	27.6 ± 7.5	<0.001	<0.001	1.000	<0.001
25(OH)D in the second trimester (ng/mL)	7.3 ± 2.2	26.5 ± 5.8	28.2 ± 6.8	<0.001	<0.001	<0.001	0.934

Data are presented in mean ± SD or number of cases (percentage); BMI, body mass index; GA, gestational age; 25(OH)D, 25-hydroxy-vitamin D;

^a^among 3 groups;

^b^between DD group and SS group;

^c^between DD group and DS group;

^d^between DS group and SS group

**Fig 2:**
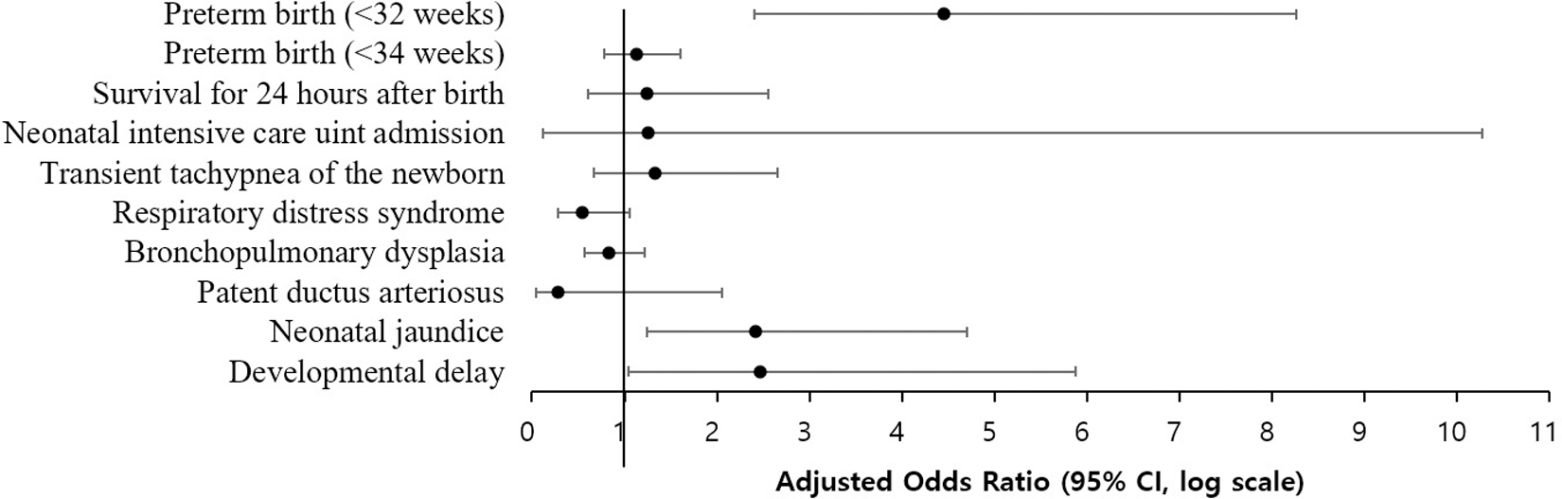
Adjusted odds ratios for various outcomes in the DD group compared to the SS group.

Table 2 presents a comparison of obstetric outcomes among the three groups, revealing no significant differences in the incidence of gestational hypertension, diabetes, and placenta previa. For delivery outcomes, including birth weight, 5-minute Apgar scores below 7, and survival within the first 24 hours after birth, only deliveries at ≥ 23 + 0 weeks of gestation were analyzed, with sample sizes of 259, 108, and 1792 for the DD, DS, and SS groups, respectively. Cesarean delivery rates were higher in the SS group (73.1%) than the DD (55.0%) and DS (50.0%) groups (p < 0.001). After adjusting for maternal age, pre-pregnancy BMI, parity, and method of conception, the DD group had significantly higher aORs for preterm birth before 32 and 34 weeks compared to the SS group (aOR = 2.47, 95%CI [1.04–5.88], p = 0.042; aOR = 2.42, 95%CI [1.24–4.71], p = 0.010, respectively) (Fig 2). However, no statistically significant difference was found between the DD and DS groups (aOR = 1.62, 95%CI [0.31–8.42], p = 0.182; aOR = 2.91, 95%CI [0.61–13.90], p = 0.182, respectively).

**Table 2:** Obstetric outcomes.

	DD group (N = 260)	DS group (N = 108)	SS group (N = 1820)	*p*-value	Adjusted OR (95% CI)^a^ (in DD group; reference: SS group)	*p*-value	Adjusted OR (95% CI)^a^ (in DD group; reference: DS group)	*p*-value
Preeclampsia	14 (5.4)	3 (3.8)	98 (5.4)	0.496	0.93 (0.47-1.83)	0.825	0.97 (0.91-1.03)	0.295
GDM	15 (5.8)	8 (7.4)	133 (7.3)	0.662	0.72 (0.37-1.42)	0.344	0.55 (0.20-1.49)	0.239
Placenta previa	4 (1.5)	0 (0.0)	35 (1.9)	0.324	1.23 (0.43-3.55)	0.699		
Delivery ≥ 23^ + 0^ week	(N = 259)	(N = 108)	(N = 1792)					
GA at delivery (week)	37.9 ± 2.8	38.7 ± 1.8	38.0 ± 1.8	<0.001				
Cesarean delivery	145 (55.0)	54 (50.0)	1310 (73.1)	<0.001	0.69 (0.50-0.96)	0.027	1.25 (0.73-2.12)	0.416
Preterm birth								
23^ + 0^–27^ + 6^ week	4 (1.5)	1 (0.9)	5 (0.3)	0.015	4.49 (0.79-25.65)	0.091	0.86 (0.07-10.0)	0.905
23^ + 0^–31^ + 6^ week	12 (4.6)	2 (1.9)	25 (1.4)	0.001	2.47 (1.04-5.88)	0.042	1.62 (0.31-8.42)	0.564
23^ + 0^–33^ + 6^ week	18 (6.9)	2 (1.9)	46 (2.6)	<0.001	2.42 (1.24-4.71)	0.010	2.91 (0.61-13.9)	0.182

Data are presented in mean ± SD or number of cases (percentage); GDM, gestational diabetes mellitus; GA, gestational age;

^a^All outcomes were adjusted for maternal age, body mass index before pregnancy, nulliparity, and method of conception.

In terms of birth outcomes, there were significant differences in birth weight among the groups, with mean weights of 2964 ± 616 g in the DD group, 3157 ± 475 g in the DS group, and 2995 ± 517 g in the SS group (p = 0.004). However, no significant differences were observed among the three groups in the rates of 5-minute Apgar scores below 7 or survival within the first 24 hours after birth. Neonatal intensive care unit (NICU) admission rates and short-term neonatal morbidities were analyzed only for neonates who survived beyond the first 24 hours, resulting in sample sizes of 257, 108, and 1782 for the DD, DS, and SS groups, respectively. NICU admission rates were higher in the SS group (23.0%) compared to the DD group (20.2%) and DS group (13.0%) (p = 0.039). Yet, after adjusting for variables like maternal age, pre-pregnancy BMI, parity, and method of conception, the aOR (95% CI) for NICU admission in the DD group versus the SS group was 0.83 (0.57–1.22), with a p-value of 0.345, indicating no statistical significance.

For short-term neonatal morbidities, comparisons of TTN, RDS, BPD, PDA, neonatal jaundice, NEC, and ICH showed a significant association only for NEC. After adjusting for maternal age, the aOR (95% CI) for NEC in the DD group, using the SS group as a reference, was 22.26 (4.16–119.3), p < 0.001. No significant differences were observed when comparing the DD group to the DS group. In addition, the incidence of RDS and NICU admissions was notably higher in the DD group compared to the SS group, although these did not reach statistical significance. Neonatal hypocalcemia did not show significant differences among the groups. While NEC was observed in a small number of cases, its occurrence was most prominent in the DD group. These findings suggest a potential link between maternal vitamin D deficiency and adverse neonatal outcomes, as detailed in Table 3.

**Table 3:** The effect of vitamin D deficiency on perinatal outcomes and postnatal morbidities.

	DD group (N = 259)	DS group (N = 108)	SS group (N = 1792)	*p*-value	Adjusted OR (95% CI)^a^ (in DD group; reference: SS group)	*p*-value	Adjusted OR (95% CI)^a^ (in DD group; reference: DS group)	*p*-value
Birth weight (gram)	2964 ± 616	3157 ± 475	2995 ± 517	0.004				
Apgar score at 5min < 7	3 (1.2)	2 (1.9)	25 (1.4)	0.874				
Survival for 24 hours after birth	257 (99.2)	108 (100.0)	1782 (97.9)	0.197	0.28 (0.04-2.06)	0.211		
NICU admission	52/257 (20.2)	14/108 (13.0)	409/1782 (23.0)	0.039	0.83 (0.57-1.22)	0.345	1.58 (0.76-3.25)	0.219
NICU hospitalization (days)	19.2 ± 25.3	15.7 ± 14.1	17.3 ± 17.0	<0.001				
Short-term morbidities	(N = 257)	(N = 108)	(N = 1782)					
TTN	11 (4.3)	2 (1.9)	155 (8.7)	0.003	0.55 (0.28-1.06)	0.073	4.77 (0.59-38.33)	0.141
RDS	15 (5.8)	2 (1.9)	71 (4.0)	0.181	1.33 (0.67-2.65)	0.421	2.46 (0.51-11.83)	0.266
BPD	15 (5.8)	3 (2.8)	24 (1.3)	0.019	1.25 (0.15-10.28)	0.833	0.42 (0.25-7.01)	0.545
PDA	12 (4.7)	4 (3.7)	61 (3.4)	0.603	1.24 (0.60-2.56)	0.565	1.09 (0.32-3.68)	0.888
Neonatal jaundice	66 (25.7)	19 (17.6)	380 (21.3)	0.164	1.13 (0.79-1.61)	0.513	1.26 (0.66-2.40)	0.478
NEC	8 (3.1)	1 (0.9)	3 (0.2)	<0.001	22.26 (4.16-119.3)	<0.001	2.23 (0.25-19.64)	0.469
ICH	3 (1.2)	1 (0.9)	6 (0.3)	0.515				
Long-term morbidity	(N = 222)	(N = 103)	(N = 1346)					
Developmental delay	23 (10.4)	7 (6.8)	42 (3.1)	<0.001	4.46 (2.41-8.27)	<0.001	1.98 (0.69-5.71)	0.207

Data are presented in mean ± SD or number of cases (percentage); NICU, neonatal intensive care unit; TTN, transient tachypnea of the newborn; RDS, respiratory distress syndrome; BPD, bronchopulmonary dysplasia; PDA, patent ductus arteriosus; NEC, necrotizing enterocolitis; ICH, intracerebral hemorrhage;

^a^All outcomes were adjusted for maternal age, body mass index before pregnancy, nulliparity, and method of conception.

Lastly, long-term neonatal outcomes were assessed after one year of corrected age, with follow-up data available for 222, 103, and 1346 cases in the DD, DS, and SS groups, respectively. The risk of developmental delay was significantly higher in the DD group compared to the SS group, with an aOR of 4.46 (95% CI: 2.41–8.27, p < 0.001), after adjusting for maternal age, pre-pregnancy BMI, parity, and method of conception. Notably, these rates remained elevated even after early correction of vitamin D deficiency. (Table 3, Fig 2).

### Principal findings

Our findings suggest that maternal vitamin D deficiency in early pregnancy is associated with increased risks of preterm birth and long-term developmental delays in offspring. Notably, even when vitamin D deficiency was corrected by mid-pregnancy, the risk of adverse outcomes remained elevated, indicating that early vitamin D levels may play a critical role in fetal and placental development.

### Review in the context of what is known

Vitamin D plays a crucial role in early pregnancy, influencing various physiological processes, including the formation and development of the placenta [10,14]. Trophoblast cells are critical in the early stages of placental development. These cells undergo proliferation and differentiation processes to form the placenta’s structural and functional components [17]. Vitamin D receptors are present in trophoblast cells, indicating that these cells are responsive to vitamin D [18]. Additionally, vitamin D has been implicated in promoting angiogenesis, the formation of new blood vessels, ensuring adequate blood supply for the development and function of the placenta [19]. Furthermore, vitamin D’s immunomodulatory properties contribute to establishing immune tolerance at the maternal-fetal interface during early pregnancy [20]. This immunomodulation is crucial for preventing rejection of the developing fetus by the maternal immune system, allowing successful placental formation. Decreased trophoblast cell proliferation and differentiation, impaired angiogenesis, and unstable immunomodulation can eventually lead to chronic sterile inflammation in the maternal-fetal face, potentially causing preterm labor and birth [21,22]. Our study found that maternal vitamin D deficiency at early pregnancy was associated with preterm birth 32 and before 34 weeks.

Vitamin D plays a key role in calcium homeostasis, and calcium is essential for various cellular processes, including those involved in placental formation [1,17]. Proper calcium levels are crucial for the structural integrity of the placenta and for supporting the transport of nutrients across the placental barrier [22]. Vitamin D also regulates the expression of numerous genes involved in cellular proliferation, differentiation, and immune response [23,24]. The modulation of gene expression by vitamin D is likely to influence the intricate processes involved in the early placental development [25]. Considering these research results, maternal Vitamin D deficiency may be linked to fetal malformations and immature development, as indicated by our findings associating vitamin D deficiency with NEC and developmental delay in babies. While the exact mechanisms are not fully understood, it is strongly suggested that vitamin D deficiency may disrupt normal fetal organ development [3,14,26]. Future studies should delve into these mechanisms further.

In our study, maternal vitamin D deficiency in the first trimester appeared to significantly impact outcomes, being associated with early preterm birth (before 34 weeks) and long-term developmental delay, both of which were challenging to prevent even with normalized 25(OH)D levels in the second trimester through intervention. This underscores the vital role of vitamin D in the early stages of rapid structural development of both the placenta and fetus.

Pregnancy induces notable changes in vitamin D metabolism, including increased placental production of calcitriol (the active form of vitamin D), enhanced intestinal calcium absorption, and elevated levels of vitamin D-binding protein, all contributing to altered circulating vitamin D levels. These changes are primarily driven by the increased demand for calcium, which is critical for placental and fetal development, and the maternal adaptations to support the growing fetus [26]. Therefore, preconception care should include screening for vitamin D deficiency to identify and address potential deficiencies before pregnancy. This proactive approach may help optimize maternal and fetal well-being, as our findings suggest that normalizing vitamin D levels after the first trimester may not fully mitigate the associated risks.

Our study revealed notable differences in perinatal and long-term outcomes among the three groups—those consistently sufficient in vitamin D (SS), those deficient throughout both trimesters (DD), and those who improved from deficiency to sufficiency (DS). The significantly higher risk of preterm birth before 32 and 34 weeks and increased developmental delays in the DD group compared to the SS group underscores the importance of maintaining adequate vitamin D levels throughout pregnancy. These differences likely stem from the role of vitamin D in early placental development, particularly in regulating trophoblast function, angiogenesis, and immune modulation. Persistent vitamin D deficiency may impair these processes, leading to chronic inflammation at the maternal-fetal interface, which could trigger preterm labor and fetal growth restriction.

Interestingly, while the DS group experienced some improvement in vitamin D status by the second trimester, their outcomes did not differ significantly from those of the DD group, particularly concerning preterm birth and developmental delays. This suggests that the timing of vitamin D correction plays a crucial role. By the time intervention improved 25(OH)D levels, critical phases of placental development may have already passed, limiting the potential to reverse any damage caused by early vitamin D deficiency. Early placental insufficiency might create a “developmental imprint,” setting the stage for adverse outcomes despite later normalization of vitamin D levels.

One notable finding was the lower NICU admission rate in the DS group compared to the DD group, albeit not statistically significant after adjustment. This could reflect the subtle benefits of mid-pregnancy vitamin D normalization, potentially supporting fetal growth and reducing the severity of certain neonatal complications. Although it may not fully counteract the risks established early in pregnancy, mid-pregnancy correction might mitigate some downstream effects, particularly those tied to late fetal growth and immune function.

Paradoxically, the SS group exhibited higher NICU admission rates compared to both the DD and DS groups. This could be attributed to the higher prevalence of pregnancies conceived through IVF within the SS group. IVF pregnancies are independently associated with increased risks of preterm birth and NICU admission due to multiple factors, including underlying infertility issues, embryo transfer procedures, and higher rates of obstetric interventions. Additionally, women undergoing IVF often receive more comprehensive prenatal care, which may include routine screening for vitamin D levels. This could partly explain why a larger proportion of the SS group had sufficient vitamin D levels, as their deficiencies may have been identified and addressed earlier during fertility treatment.

This study establishes a connection between deficient vitamin D levels in the first trimester and increased risks of adverse perinatal and long-term developmental outcomes, implying that subsequent normalization of vitamin D status may not mitigate these risks. These results emphasize the potential importance of recommending vitamin D screening and intervention before pregnancy as essential elements of routine preconception care to improve maternal and child health.

### Clinical applications

Our findings highlight the critical role of maintaining sufficient vitamin D levels from early pregnancy. Maternal vitamin D deficiency in the first trimester is linked to adverse perinatal and long-term developmental outcomes. Notably, later correction of vitamin D levels in the second trimester did not fully mitigate these risks, emphasizing the importance of early intervention.

We recommend incorporating pre-pregnancy vitamin D screening and intervention into routine preconception care to optimize maternal vitamin D levels before conception. Additionally, early prenatal vitamin D monitoring during the first trimester is crucial to identify and address any deficiencies as soon as possible. Tailored supplementation strategies should also be considered, taking into account individual variations in vitamin D metabolism and absorption. While prenatal vitamin D monitoring remains essential, these recommendations underscore the importance of placing even greater emphasis on pre-pregnancy screening to prevent early deficiencies and their potential consequences.

### Strengths and limitations

The strength of this study lies in the consistent analysis of data over approximately 6 years, conducted within a single medical center. Notably, to the best of our knowledge, it is the first study that examines the impact of maternal vitamin D deficiency during both the first and second trimesters on offspring’s prognosis, including long-term neurodevelopmental status. In particular, there are no studies on pregnancy outcomes in cases where maternal 25(OH)D levels were low in early pregnancy but improved after the second trimester.

However, it’s essential to acknowledge several limitations. This study concentrated on pregnant women who underwent 25(OH)D blood tests during the first and second trimesters, omitting cases of miscarriage in early pregnancy or terminations due to fetal abnormalities.

Another limitation of our study is the unequal sample sizes among the groups, with 260 in the DD group, 108 in the DS group, and 1820 in the SS group. We acknowledge that these discrepancies could potentially affect the statistical power of our analyses. To mitigate this, we employed multivariable logistic regression models adjusted for maternal age, pre-pregnancy BMI, parity, and method of conception, which allowed us to control for confounding factors and obtain more reliable effect estimates. Additionally, we presented adjusted odds ratios (aORs) with 95% confidence intervals to provide a clearer interpretation of the associations. While the smaller sample size of the DS group may have limited our ability to detect certain associations, the consistent and significant findings observed in the DD group strengthen the validity of our conclusions. Furthermore, because this study was conducted among women from a monocultural background who tend to avoid sunlight exposure, the number of women exhibiting sufficient maternal 25(OH)D levels, considered to be ≥ 40 ng/mL as suggested by several studies [3], was very low, making it impractical to analyze them as a separate group. Moreover, the retrospective design presents challenges, with some cases lost during extended follow-up due to relocation or transfer to other clinics, leading to a lack of neonatal developmental outcomes in the study. To mitigate these limitations, future studies should contemplate adopting a prospective approach. Lastly, a limitation arises from the observational nature of this study, which restricts our ability to fully control for pregnant women’s lifestyle behaviors—such as their intake of vitamin D-rich foods, sun exposure, and physical activity—despite these factors potentially influencing maternal vitamin D levels. However, we believe this does not detract from our study’s primary focus, which is not centered on the normalization of low vitamin D levels during early pregnancy but rather on the potential consequences of early deficiencies.

## Conclusion

In this study, maternal low first-trimester vitamin D levels were associated with elevated risks of preterm birth and neurodevelopmental delay in offspring, indicating that subsequent normalization of vitamin D status may not alleviate these risks. These findings underscore the potential for recommending vitamin D screening and optimization before pregnancy as integral components of routine preconception care to enhance maternal and child health.
